# Sugar‐Sweetened Beverages Taxation Plan in Indonesia: Call for Political Commitment

**DOI:** 10.1002/puh2.217

**Published:** 2024-07-15

**Authors:** Risyad Abiyyu Siregar, Fona Qorina, Ayers Gilberth Ivano Kalaij, Hera Afidjati, Dhani Latifani, Muhammad Faisal Putro Utomo, Louisa Patricia Sophia, Azizah Salsabila Mahmud

**Affiliations:** ^1^ School of Public Health Imperial College London London UK; ^2^ Evidence‐Based Health Policy Center Indonesian Medical Education and Research Institute Faculty of Medicine Universitas Indonesia Jakarta Indonesia; ^3^ Faculty of Public Health Universitas Indonesia Jakarta Indonesia; ^4^ Bhayangkara Tk.I R. Said Sukanto Police Hospital Jakarta Indonesia; ^5^ Faculty of Health Law Soegijapranata Catholic University Semarang Indonesia; ^6^ Faculty of Medicine Universitas Pembangunan Nasional “Veteran” Jakarta Jakarta Indonesia; ^7^ Departement of Nutrition Faculty of Medicine, Universitas Indonesia ‐ Dr. Cipto Mangunkusumo General Hospital Jakarta Indonesia

**Keywords:** excise tax, Indonesia, policy implementation, sugar‐sweetened beverages

## Abstract

Indonesia is grappling with a rise in obesity and diabetes, partially driven by a high consumption of sugar‐sweetened beverages. The prevalence of obesity among adults more than doubled from 2007 to 2018, and diabetes rates have also increased. In response, the Indonesian government has proposed an excise tax on sugar‐sweetened beverages to reduce consumption. However, the implementation of this policy has repeatedly been delayed, with the latest postponement to 2024. Sugar‐sweetened beverages contribute significantly to chronic diseases, increasing the healthcare burden and reducing economic productivity. Taxation, a widely used public health strategy in the form of fiscal measures, can decrease consumption by raising prices, raising public awareness, encouraging product reformulation, and generating revenue. Despite its potential benefits, the proposed tax in Indonesia faces substantial political and commercial challenges. The president‐elect Prabowo Subanto has not explicitly supported the tax in his political manifesto, raising concerns about further delays due to industry lobbying. Successful implementation of the tax requires robust political will, public and civil society pressure, and effective cross‐sector cooperation. The government must ensure clear policy goals, equal application to domestic and foreign products, and supportive measures such as providing free drinking water alternatives. Transparent stakeholder consultations can build broad‐based support, whereas effective monitoring and evaluation frameworks are essential for compliance. To gain public support, allocating tax revenues to public health and social programs is crucial. Although initial resistance is expected, strong enforcement mechanisms can ensure adherence. With determined political commitment and public advocacy, the sugar‐sweetened beverage tax can significantly reduce obesity and diabetes rates, improving public health outcomes and economic productivity in Indonesia. This commentary provides an overview of the proposed tax, explores its challenges, and offers recommendations for successful implementation.

## 1 Introduction

There has been a stable rise in obesity and its related diseases in Indonesia. The prevalence of obesity among adults has increased more than twofold, from 10.5% (2007) to 21.8% (2018) [1]. Meanwhile, the prevalence of diabetes mellitus among adults above 15 years of age has increased from 6.9% to 8.5% [1]. The consumption of sugar‐sweetened beverages may play a role in this phenomenon. A study by Ferretti and Mariani reported that Indonesia has the third highest per capita consumption of sugar‐sweetened beverages in the Southeast Asia region, with 20.23 L/person/year [2]. At least 61% of Indonesians consume sugar‐sweetened beverages daily, with the highest consumer among the 3–4‐year‐old age group (68.6%) [3].

To overcome this problem, the Indonesian government planned to tax sugar‐sweetened beverages to reduce consumption. However, the implementation of this policy has been delayed several times. Lately, the Indonesian Ministry of Finance (MoF) has included sugar‐sweetened beverages taxation as one of the sources of income in the 2023 national budget. However, this was eventually repealed and delayed to the 2024 national budget [4].

There have been concerns that this policy could be further delayed. As of 18 March 2024, the government has yet to release the details of the excise tax plan. With policies that address the commercial determinants of health having a track record of delay and even repealed, the political commitment from the incumbent (and successive) government is needed more than ever. This commentary shall give a general overview of the sugar‐sweetened beverages taxation plan in Indonesia, its challenges, and recommendations to successfully implement the policy.

## 2 Burden of High Sugar Intake‐Related Diseases in Indonesia

It is well known that sugar‐sweetened beverage consumption is tightly associated with an increased risk of chronic diseases. Sugar‐sweetened beverages contain calories in liquid form that might not give similar satiation compared to solid food, which incites excessive consumption [5]. This can lead to obesity which is itself a risk factor for cardiometabolic diseases such as insulin resistance, hypertension, and dyslipidemia. This is added by the fact that dietary risks and high body mass index (BMI) are the third and fifth leading risk factors for disability‐adjusted life years (DALYs) in Indonesia, respectively [6].

High consumption of sugary drinks, which coincides with an increasing burden of obesity‐related diseases, poses a significant public health threat to Indonesian people. The decrease in life expectancy and the increase in years lived with disability (YLDs) lower productivity and endanger the working population's ability to earn a living. It is worth noting that this situation can increase the financial burden on the Indonesian National Health Insurance System, because approximately IDR 14.3 trillion (equivalent to USD 892 million in the exchange rate of May 26, 2024) or 14.7% of the total health costs for secondary and tertiary healthcare are being spent on treating cardiometabolic diseases [7].

## 3 Taxing Sugar‐Sweetened Beverages

Taxation as a public health intervention is not a novel invention. What experts call “fiscal measures for health” is rooted in the taxation of goods such as tobacco and alcohol. Currently, over 50 countries around the world have implemented sugar‐sweetened beverages taxation with varying degrees [8]. World Health Organization have recommended sugar‐sweetened beverages taxation as one of the “best buys” to tackle noncommunicable diseases (NCDs) worldwide [9].

Implementing an excise tax on sugar‐sweetened beverages can alter the sugar‐sweetened beverage consumption among the public in numerous ways. The World Bank [10] proposed four main mechanisms in which sugar‐sweetened beverages taxation works (1) by increasing retail prices, (2) by raising public awareness, (3) by encouraging the sugar‐sweetened beverages industry to reformulate their product, and (4) by generating revenue for the country. Increasing the retail price is believed to be the ultimate aim of taxing sugar‐sweetened beverages. By increasing the price, sugar‐sweetened beverage consumption patterns are hoped to decrease. A systematic review of sugar‐sweetened beverages taxation in middle‐income countries concluded that this policy did decrease sugar‐sweetened beverages consumption, with lower socioeconomic groups more affected by these price changes [11]. According to an economic model projecting changes in demand for sugar‐sweetened beverages after taxation in Indonesia, a 20% increase in the price of sugar‐sweetened beverages could lead to an average reduction in demand of 17.5% (95% CI 14.3%−18.6%) [12].

## 4 Sugar‐Sweetened Beverages Taxation in Indonesia: Progress and Challenges

The proposal to implement a tax on sugar‐sweetened beverages has been repeatedly raised within the governmental sphere but has yet to materialize. The MoF initially attempted to introduce an excise tax on sugar‐sweetened beverages in 2012, only to face rejection by the Commission XI of the National House of Representatives. Subsequent efforts in 2015 were short‐lived, as the measure was later repealed in the 2016 budget. Following a period of dormancy, the issue resurfaced in early 2020. The MoF initially included sugar‐sweetened beverages as a source of excise revenue in the 2023 national budget, only to be postponed again to the 2024 budget cycle [13].

As of the current budget cycle, there have been no indications of revisions to the sugar‐sweetened beverages excise plan. The MoF has indicated its intention to convene coordination meetings with various ministries to deliberate the implementation of this policy, potentially paving the way for its enactment [14]. However, new challenges have emerged with the advent of the new government following recent elections.

Policies have always been inseparable from the political will of the leader of the country which decides the objectives and policymaking pattern [15]. The president's political agenda could be the drive of novel regulations and policies. The General Election Commission has declared Prabowo Subianto, accompanied by his running mate Gibran Rakabuming, as the triumphant victors of the 2024 presidential election, securing a landslide victory with over a half of the total votes cast [16]. Despite their assurances to uphold the agendas of the preceding (Joko Widodo) administration, concerns persist regarding the realization of the proposed policy. Notably, their campaign manifesto did not mention sugar‐sweetened beverages taxation or broader health‐related fiscal measures [17], further contributing to the prevailing uncertainties surrounding the policy's fate.

Public health policies that encumber commercial interests often encounter delays or face repeal, owing to robust lobbying efforts directed at political stakeholders by affected industries. An illustrative example is the repeal of the Minimum Unit Pricing (MUP) plan for alcoholic beverages in the United Kingdom [18], purportedly influenced by intense lobbying from the alcohol industry. Similarly, the postponement of the implementation of the High Fat Salt and Sugar (HFSS) food advertising ban on UK television from October 2022 to October 2025 underscores the influence of industry lobbying on policy timelines [19].

## 5 Next Steps

Given the anticipated lobbying efforts from affected industries, the realization of the sugar‐sweetened beverages taxation plan hinges upon robust political will. It is imperative for civil society organizations and the general public to exert pressure on the government to ensure the policy's implementation as scheduled and to remain vigilant against potential impediments. Furthermore, the government must carefully deliberate on the most effective and feasible form of sugar‐sweetened beverage taxation, one that optimally serves the health and economic interests of the Indonesian populace.

The sugar‐sweetened beverages excise implementation strategy includes several stages, namely, (1) considering the policy design, (2) discussing and promoting the policy, (3) getting support for implementing the policy, (4) enforcing the policy, and (5) monitoring and evaluating the policy. Determining goals and targeted products must be clear to avoid mistakes, facilitate the identification of potential substitute products, and facilitate policy monitoring and evaluation. To avoid international‐level trade disputes, it is recommended that sugar‐sweetened beverages excise duties be applied equally to domestic and foreign products. It is necessary to ensure that there are supporting policies as an alternative to sugar‐sweetened beverages consumption, for example, by providing easy access to safe and free drinking water through ready‐to‐drink water taps in schools and public spaces. Discussion and consultation with various stakeholders are expected to protect against conflicts of interest and provide transparent access in the policy development process. It is important to develop cross‐sector cooperation supported by strong political leadership so that its implementation can be effective. This discussion and cooperation will be stronger if it is based on evidence of positive impacts on the economy and health. It is hoped that the allocation of excise revenue from sugar‐sweetened beverages to public health and social programs will increase public support for the implementation of the sugar‐sweetened beverages tax policy.

Although initial resistance to this policy is anticipated, the government must establish robust enforcement mechanisms to ensure compliance with sugar‐sweetened beverages excise regulations. Such mechanisms should encompass both incentives to reward compliance and sanctions to deter noncompliance effectively. Consequently, the development of efficient monitoring and evaluation frameworks, involving pertinent regulatory bodies, is imperative to uphold the integrity of the policy.

## 6 Conclusion

In conclusion, the imposition of taxes on sugar‐sweetened beverages represents an effective strategy for curbing public consumption and addressing public health concerns. The recent advancements in Indonesia's taxation policy signal a strong potential for enactment, despite historical setbacks. However, the advent of a new governmental regime introduces uncertainty, potentially hindering progress or leading to policy regression. The success of this initiative hinges on unwavering political commitment, complemented by vigilant oversight from the public and civil society organizations to ensure transparent and accountable policymaking.

## Author Contributions

**Risyad Abiyyu Siregar**: Conceptualization (lead); writing – original draft (equal); formal analysis (equal); writing – review and editing (lead). **Fona Qorina**: writing – original draft (equal); formal analysis (equal). **Ayers Gilberth Ivano Kalaij**: writing – original draft (equal); formal analysis (equal); writing – review and editing (supporting). **Hera Afidjati**: Conceptualization (supporting); writing – original draft (equal). **Dhani Latifani**: writing – original draft (equal). **Muhammad Faisal Putro Utomo**: writing – original draft (equal). **Louisa Patricia Sophia**: Conceptualization (supporting); writing – original draft (equal). **Azizah Salsabila Mahmud**: Conceptualization (supporting); writing – original draft (equal).

## Conflicts of Interest

The authors declare no conflicts of interest.

## Data Availability

The authors have nothing to report.
